# Effects of Megaplasmid Loss on Growth of Neurotoxigenic *Clostridium butyricum* Strains and Botulinum Neurotoxin Type E Expression

**DOI:** 10.3389/fmicb.2016.00217

**Published:** 2016-02-25

**Authors:** Concetta Scalfaro, Angelo Iacobino, Laura Grande, Stefano Morabito, Giovanna Franciosa

**Affiliations:** ^1^Unit of Foodborne Zoonoses, Department of Food Safety and Veterinary Public Health, Istituto Superiore di SanitàRome, Italy; ^2^Unit of Systemic Bacterial Infections, Department of Infectious, Parasitic and Immune-Mediated Diseases, Istituto Superiore di SanitàRome, Italy

**Keywords:** neurotoxigenic *Clostridium butyricum* type E, megaplasmid, plasmid curing, microbial growth, botulinum neurotoxin expression, PFGE, RTq-PCR

## Abstract

*Clostridium butyricum* strains that atypically produce the botulinum neurotoxin type E (BoNT/E) possess a megaplasmid of unknown functions in their genome. In this study, we cured two botulinum neurotoxigenic *C. butyricum* type E strains of their megaplasmids, and compared the obtained megaplasmid-cured strains to their respective wild-type parental strains. Our results showed that the megaplasmids do not confer beta-lactam resistance on the neurotoxigenic *C. butyricum* type E strains, although they carry several putative beta-lactamase genes. Instead, we found that the megaplasmids are essential for growth of the neurotoxigenic *C. butyricum* type E strains at the relatively low temperature of 15°C, and are also relevant for growth of strains under limiting pH and salinity conditions, as well as under favorable environmental conditions. Moreover, the presence of the megaplasmids was associated with increased transcript levels of the gene encoding BoNT/E in the *C. butyricum* type E strains, indicating that the megaplasmids likely contain transcriptional regulators. However, the levels of BoNT/E in the supernatants of the cured and uncured strains were similar after 24 and 48 h culture, suggesting that expression of BoNT/E in the *C. butyricum* type E strains is not ultimately controlled by the megaplasmids. Together, our results reveal that the *C. butyricum* type E megaplasmids exert pleiotropic effects on the growth of their microbial hosts under optimal and limiting environmental conditions, and also highlight the possibility of original regulatory mechanisms controlling the expression of BoNT/E.

## Introduction

Microorganisms of the *Clostridium butyricum* species have been attracting increasing interest as they produce butyric acid which has many chemical, pharmaceutical and biotechnological applications, and are capable of converting glycerol to 1,3-propanediol, a monomer of the production of plastics (Szymanowska-Powałowska et al., 2014). Moreover, some *C. butyricum* strains have been shown to exert health benefits in humans and animals and are therefore used as probiotics (Shinnoh et al., 2013; Zhao et al., 2013).

However, certain *C. butyricum* strains produce the botulinum neurotoxin (BoNT), which is one of the most toxic proteins to humans and some vertebrate animals (Rossetto et al., 2014). BoNT inhibits acetylcholine-mediated nerve-to-muscle transmission thus leading to the flaccid paralysis typical of botulism, a rare disease in humans that is mainly caused by the ingestion of BoNT in contaminated food (foodborne botulism), or by direct absorption of BoNT from the intestine or a wound infected with BoNT-producing microorganisms (intestinal toxemia botulism in infants or adults, and wound botulism, respectively; Sobel, 2005).

Seven (A to G) BoNT serotypes sharing similar structures and functions have long been recognized, based on specific neutralization with distinct polyclonal antibodies (Hatheway, 1992), although a novel “hybrid” BoNT has recently been described that is neutralized by existing antibodies (Barash and Arnon, 2014; Maslanka et al., 2015). Except for BoNT/G, which is produced by the rare *C. argentinense* species, the other BoNT serotypes are formed by heterogeneous microorganisms conventionally grouped within the *C. botulinum* species; BoNT/E and BoNT/F can also be produced by sporadic strains of the typically non-neurotoxigenic *C. butyricum* and *C. baratii* species, respectively (Hatheway, 1992).

Neurotoxigenic *C. butyricum* type E strains have been isolated from cases of intestinal toxemia botulism in infants and adults in Italy, Japan, USA, Ireland, and UK (Aureli et al., 1986; Fenicia et al., 1999, 2002; Abe et al., 2008; Dykes et al., 2015; Shelley et al., 2015); foodborne botulism cases in China, India and Italy (Meng et al., 1997; Chaudhry et al., 1998; Anniballi et al., 2002); food and environmental samples (soil and lake shore) in China (Meng et al., 1997, 1999); and, more recently, from pets (turtles) which were identified as the likely source of infection in two infant botulism cases in Ireland (Shelley et al., 2015).

By comparing the genome sequences of a neurotoxigenic *C. butyricum* type E strain and two *C. botulinum* type E strains, Hill et al. noted that the *bont/e* gene cluster – consisting of the gene encoding BoNT/E and some accessory genes encoding non-toxic proteins that assemble to BoNT/E to yield a toxin complex – was inserted into the same chromosomal resolvase (*rarA*) operon (Hill et al., 2009). Interestingly, all the inserted sequences contained an intact *rarA* gene replacing the split *rarA* gene: since resolvase is involved in the mobilization of transposons, transposon-mediated horizontal gene transfer was hypothesized to be responsible for the presence of the *bont/e* gene cluster in the *C. botulinum* and *C. butyricum* species (Hill et al., 2009). The subsequent finding that the *bont/e* gene cluster is carried by plasmids in several *C. botulinum* type E strains supports that it can reside within a variety of potentially mobile genetic elements (Zhang et al., 2013).

We have recently shown that ten distinct neurotoxigenic *C. butyricum* type E strains from Italy and China, all possessing a chromosomal *bont/e* gene, share a very large plasmid of different sizes in their genome: specifically, two megaplasmids approximately 590 and 758 kb in size were identified among the six Italian strains analyzed (Franciosa et al., 2011; Iacobino et al., 2013). Given the high cost for the carriage and maintenance of such megaplasmids, their consistent presence among the strains could indicate that they play a crucial role in host physiology and pathogenesis.

In this study, we cured two neurotoxigenic *C. butyricum* type E strains from Italy of their differently sized megaplasmids, and compared the wild-type (WT) parental strains with the megaplasmid-cured (Δmp) derivative strains in order to study the megaplasmid function(s).

## Materials and Methods

### *Clostridium butyricum* Strains and Culture Conditions

Both neurotoxigenic *C. butyricum* type E strains used in this study were from Italy. Strain ISS-21 was one of the isolates from infant botulism cases that was first recognized as BoNT/E-producing *C. butyricum* in 1985 (Aureli et al., 1986). Strain ISS-190 was recovered from another infant botulism case that occurred in 2001 (Fenicia et al., 2002). Strains ISS-21 and ISS-190 possess megaplasmids that are 590 kb (*mp590*) and 758 kb (*mp758*) in size, respectively, and they both carry an additional smaller plasmid of ∼9 kb. The two strains have previously been shown to be clonally related, with most of the genetic diversity between the strains consisting of a ∼168 kb genetic region that is present in the larger megaplasmid of strain ISS-190 and totally missing from the smaller megaplasmid of strain ISS-21 (Iacobino et al., 2013).

Clostridia strains were cultured in tryptone-peptone-glucose-yeast extract (TPGY) broth (pH 7), or in TPGY agar plates or egg yolk (EY) agar plates (Oxoid Ltd., Basingstoke, Hampshire, England), and incubated at 37°C under anaerobic conditions (Anaerogen, Oxoid; 9–13% carbon dioxide and oxygen < 1% according to the manufacturer), unless otherwise specified.

### Curing Experiments and PCR Detection of Plasmids

Strains ISS-21 and ISS-190 were sub-cultured repeatedly in fresh TPGY broth tubes (5 ml) containing acridine orange (Sigma–Aldrich, Milan, Italy) as a plasmid curing agent at increasing concentrations from 3 to 16 μg/ml. The broth cultures were incubated anaerobically at 37°C overnight or until visible growth was evident. At time intervals of two or three serial passages, the broth cultures were spread onto EY agar plates and incubated at 37°C for 48 h under anaerobic conditions. Single colonies were randomly picked from the EY agar plates and suspended in 50 μl of 1XTE buffer (10 mM Tris, pH 8.0, and 0.1 mM EDTA). Each colony suspension (5 μl) was directly used as a DNA template in separate PCR reactions to detect four distinct megaplasmid-encoded beta-lactamase genes (namely, *latt*, *bl*, *mb*, and *dp*). The colonies that were PCR negative for the *latt*, *bl*, *mb*, and *dp* genes were further analyzed by PCR for the chromosome-encoded *bont/e* gene and the *mob* gene, which is encoded by the smaller plasmid. The specific oligonucleotide primers used in the reactions and the PCR conditions were the same as reported elsewhere (Franciosa et al., 2011; Iacobino et al., 2013).

### Pulsed-Field Gel Electrophoresis and Southern Blot Experiments

Genomic DNA for pulsed-field gel electrophoresis (PFGE) analyses was extracted from the WT and Δmp derivative strains as described previously (Franciosa et al., 2011; Iacobino et al., 2013). DNA samples were digested with *Xho*I or *Sma*I (Roche Diagnostics, Milan, Italy) following the manufacturer’ instructions. The unrestricted and restricted DNA samples were loaded on 1% GellyPhor PFGE agarose gel (Euroclone, Milan, Italy); DNA isolated from *Salmonella enterica* serovar Braenderup strain H9812 and restricted with *Xba*I (Roche Diagnostics) served as the molecular standard (Hunter et al., 2005). PFGE runs were performed using a contour-clamped homogeneous electric field system (CHEF Mapper apparatus, Bio-Rad Laboratories, Hercules, CA, USA). A constant temperature of 14°C was used, and the electrophoresis parameters were as follows: voltage of 6 V/cm, an angle of 120, and switch times of 5–60 s (linear ramping factor) for 18 h to separate the unrestricted DNA samples, or 4–40 s (linear ramping factor) for 20 h to separate the restricted DNA samples. Gels were stained with ethidium bromide and visualized with a GelDoc 2000 apparatus (Bio-Rad Laboratories).

Southern hybridizations of pulsed-field gels with digoxigenin (DIG)-labeled *bont/E*, beta-lactamase (*latt*), and *mob* gene probes were carried out by using standard techniques (Franciosa et al., 2011; Iacobino et al., 2013). The hybridized probes were detected using DIG detection reagents (Roche Diagnostics) according to the manufacturer’s instructions.

### RNA Isolation, cDNA Synthesis, and Quantitative Reverse Transcription PCR

For RNA isolation, the WT and Δmp derivative strains were inoculated in TPGY broth (pH 7) and incubated anaerobically at 37°C: growth curves were generated by measuring the optical density (OD)_600_ values at appropriate time intervals. Samples were collected from the broth cultures at different time points and centrifuged at 5000 × *g* for 10 min at 4°C, and the bacterial pellets were then stored at -80°C until RNA extraction.

RNA was extracted using the Total RNA Purification Kit (Norgen Biotek Corporation, Thorold, ON, Canada). A conventional on-column DNase digestion was performed during the RNA purification protocol; to ensure elimination of all contaminating DNA, the extracted RNA samples were subjected to a second DNase treatment with the DNase – RNase free kit (Roche Diagnostics) according to the manufacturer’s instructions. The RNA concentration was determined using the Qubit Fluorometer (Invitrogen, Carlsbad, CA, USA).

cDNA samples were prepared from 100 ng of RNA using the QuantiTect Reverse Transcription kit according to the manufacturer’s instructions (Qiagen, Chatsworth, CA, USA). Negative reverse transcription (RT) controls were produced by identical reaction conditions without reverse transcriptase.

Quantitative real-time PCR (RT-qPCR) was carried out in the Rotor-Gene 6000 real-time thermal cycler (Qiagen). The primers and probes used in this study for the target *bont/e* gene and for the *16S rrn* reference constitutive gene are listed in **Table 1**. The reaction mixtures were composed of 1X SensiMix II Probe No-ROX Kit (Bioline, Paris, France), 0,5 μM each primer, 0,2 μM each probe and 2 ng cDNA, in a total volume of 20 μL.

**Table 1:** Primers and probes for the target *bont/e* gene and for the *16S rrn* reference constitutive gene.

Primer or probe	Sequence (5′→3′)	Reference
*bont/e * (target gene)		
Primers		
BE1430F	5′-GTGAATCAGCACCTGGACTTTCAG-3′	Kimura et al., 2001
BE1709R	5′-GCTGCTTGCACAGGTTTATTGA-3′	Kimura et al., 2001
Probe		
BE1571FP (Probe)	5′-6-FAM -ATGCACAGAAAGTGCCCGAAGGTGA-BHQ-1-3′	Kimura et al., 2001

*16S rrna * (reference gene)^∗^		
Primers		
But_16S1	5′- CGTGTCGTGAGATGTTGGGTTAA -3′	This work
But_16S2	5′- CGCGAGGTTGCATCTCATTGT -3′	This work
Probe		
But_16S (Probe)	5′-HEX-ACTCTAGCGAGACTGCCCGGGTT-BHQ-1-3′	This work


*^∗^16S ribosomal RNA gene (locus tag CLP_RS20835) of *Clostridium butyricum* E4 strain BL5262 (GenBank Reference Number: NZ_ACOM01000011.1).*

The cycling conditions included 10 min at 95°C, followed by 40 cycles of 95°C for 15 s and 60°C for 60 s. PCR efficiencies were determined for each primer pair based on a standard curve constructed from serial dilutions of cDNA samples. The calculated efficiencies were 0.99 for 16S *rrn* and 0.92 for *bont/e*.

All reactions were performed in triplicate using RNA samples from two independent extractions. Target gene transcript levels were normalized to the 16S *rrn* transcript level and the relative transcript changes were calculated using the 2^-ΔΔCt^ method (Livak and Schmittgen, 2001).

### Mouse Bioassay

The toxicity of BoNT/E produced by the cured and uncured *C. butyricum* strains was quantified by mouse bioassay, i.e., the most sensitive standard method for measuring active BoNTs (Singh et al., 2013).

TPGY broth cultures (24 and 48 h) of the WT and Δmp derivative strains were centrifuged at 12000 × *g* for 20 min at 4°C. The supernatants were treated with 0.5% trypsin 1:250 (BD-Difco, Milan, Italy) for 20 min at room temperature, in order to activate the BoNT/E produced by the *C. butyricum* strains as described by Kozaki et al. (1991); they were then fivefold diluted in phosphate buffer (pH 6.4) containing 0.2% gelatin. Groups of four CD1 mice (25 g weight) were injected intraperitoneally with 0.5 ml of the different dilutions per mice, and then monitored over 4 days for signs of botulism (Centers for Disease Control and Prevention, 1998). Survivals and deaths were recorded, and the results were expressed as mouse 50% lethal dose (LD50) per milliliter (Reed and Muench, 1938). The experimental protocol was approved by the Italian Ministry of Health (Authorization No. 291/2015).

### Assessment of Growth Under Limiting Salt, pH, and Temperature Conditions

The WT and Δmp derivative strains were cultured in fresh TPGY broth tubes that were either supplemented with 3% (w/v) sodium chloride (NaCl), or adjusted to a pH of 5 with 6 N HCl, in order to compare their growth under limiting salt concentrations and pH values, respectively. The TPGY broth cultures were anaerobically incubated at 37°C for 8 days.

In separate sets of experiments, the WT and Δmp derivative strains were inoculated in plain TPGY broth tubes, covered with a layer of sterile vaseline oil to produce anaerobiosis conditions, and incubated at 15°C in a recirculating refrigerated water bath (Haake G, ThermoScientific, Karlsruhe, Germany), in order to compare their growth under limiting conditions of temperature.

In all experiments, the initial clostridia inocula were adjusted to OD_600_ ∼0.2–0.3 using a spectrophotometer (Biophotometer, Eppendorf, Milan, Italy). Growth curves were generated by measuring the OD_600_ values of the broth cultures at appropriate intervals of time. All growth experiments were performed in triplicate.

### Sugar Fermentation

We evaluated the sugar fermentation patterns by using a basal medium consisting of tryptone (15 g/l), yeast extract (7 g/l), *L*-cystine (0.25 g/l), sodium chloride (2.5 g/l), ascorbic acid (0.1 g/l), sodium thioglycollate (0.5 g/l), bromothymol blue (0.01 g/l), and agar (0.75 g/l), which was supplemented with one of the following carbohydrates: glucose, maltose, mannitol, mannose, xylose, glycerol, lactose, fructose, *L*-arabinose, rhamnose, starch, sucrose, galactose, or cellobiose. Each carbohydrate was added at a final concentration of 0.6% (w/v), except for starch which was added at a final concentration of 0.25% (Dowell et al., 1977). Tubes containing each sugar fermentation medium (7 ml) were inoculated with 2% (v/v) overnight TPGY broth cultures of the WT and Δmp derivative strains, and incubated anaerobically for 48 h at 37°C. The fermentation was considered positive when the color of the indicator (bromothymol blue) turned from green to yellow upon acidification due to carbohydrate fermentation. Sugar fermentation assays were performed in duplicate.

### Sporulation Assay

Single colonies of the WT and Δmp derivative strains were picked from EY agar plates and inoculated into separate TPGY broth tubes (10 ml), which were then heat shocked at 70°C for 10 min and incubated anaerobically at 37°C for 20 days in order to allow efficient sporulation.

For spore enumeration, the broth cultures were heat treated at 70°C for 10 min to eliminate any vegetative bacterial cells; 10-fold dilutions were then prepared and plated in duplicate onto TPGY agar plates. After 48 h anaerobic incubation at 37°C the colony-forming units (CFU) were counted on the TPGY plates. When the CFU numbers were below detectable levels, the spores were enumerated by the most probable number method (USDA-FSIS, 2014). Briefly, sets of three TPGY broth tubes were inoculated with 10-fold dilutions of the heat shocked spore suspensions; the tubes were then incubated at 37°C for 48 h under anaerobic conditions. Tubes with observable turbidity after incubation were recorded as positive, and the purity of the positive cultures was checked on EY agar plates.

The mean spore numbers were determined by combining the data from three separate experiments.

### Beta-Lactamase Production

Beta-lactamase production was assayed using Beta-Lactamase Identification Sticks (Oxoid). Since beta-lactamase production can be induced in some clostridia strains upon exposure to beta-lactam antimicrobials (Hedberg and Nord, 1996), the beta-lactamase activity of the WT and Δmp derivative strains was evaluated with and without induction. For induction, strains were sequentially transferred in TPGY broth containing increasing concentrations of penicillin G or ampicillin (Sigma–Aldrich), starting from 0.062 μg/ml. The broths with the highest concentrations of beta-lactams that could support growth were streaked on TPGY agar plates containing the same beta-lactams concentrations. Colonies from the agar plates were directly assayed for beta-lactamase production. Single colonies of the beta-lactamase producing *Staphylococcus aureus* strain ATCC 29213 were used as the positive control on Brain Heart Infusion agar plates (Oxoid).

### Statistical Analyses

Student’s *t*-test was used to perform all pairwise comparisons between the WT and Δmp derivative strains. All calculations were performed with GraphPad Prism version 6 software (GraphPad Software, San Diego, CA, USA), and *p-*values < 0.05 were considered statistically significant.

## Results

### Isolation of Δmp Derivatives of the WT Neurotoxigenic *C. butyricum* Type E Strains

Single colonies that were PCR negative for the *latt*, *bl*, *mb*, and *dp* beta-lactamase genes, but positive for the *bont/e* and *mob* genes, were obtained from strains ISS-21 and ISS-190 after 11 and 24 successive transfers, respectively, in the presence of acridine orange (data not shown). Since the four selected *latt*, *bl*, *mb*, and *dp* beta-lactamase genes are located within the megaplasmids of the WT *C. butyricum* type E strains, as previously reported based on the analysis of the available draft genome sequences of two Italian *C. butyricum* type E strains (GenBank Reference Numbers: NZ_ACOM00000000.1 and NZ_ABDT00000000.1) and on the genetic maps obtained for the megaplasmids (Iacobino et al., 2013), the negative beta-lactamase PCR results suggest the loss of the megaplasmid. Conversely, the positive *bont/e* and *mob* PCR products indicate that the same colonies retained both the chromosomal *bont/e* gene and the smaller plasmid-encoded *mob* gene (Franciosa et al., 2011). These colonies were then selected as candidate Δmp derivatives of the WT parental *C. butyricum* type E strains, and megaplasmid removal in the cured candidates was verified by PFGE and Southern blot analysis.

**FIGURE 1:**
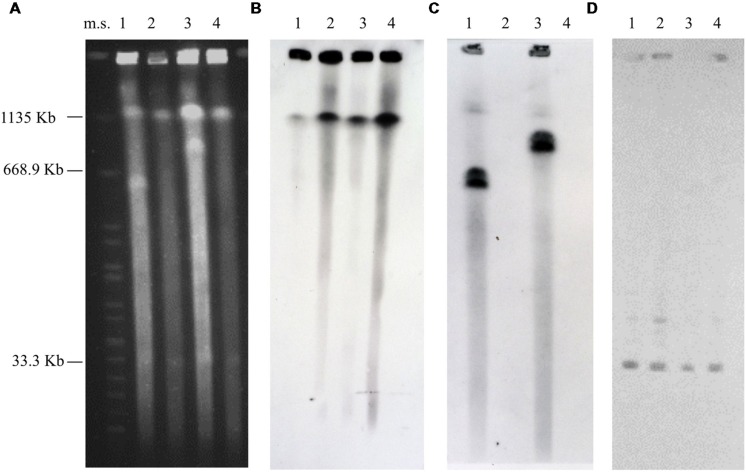
**Pulsed-field gel electrophoresis patterns of the undigested genomic DNA of the WT *Clostridium butyricum* type E strains and their Δmp derivative strains **(A)**, and Southern hybridization analyses using the *bont/E* gene probe **(B)**, the *latt* gene probe **(C)**, and the *mob* gene probe **(D)**.** Strains ISS-21 (lane 1), ISS-21Δ*mp590* (lane 2), ISS-190 (lane 3), and ISS-190Δ*mp758* (lane 4). Ms, molecular standard, *Xba*I-digested DNA from *Salmonella* Braenderup strain H9812 (Hunter et al., 2005).

**Figure 1A** shows the PFGE patterns of the undigested genomic DNA patterns of the WT and candidate Δmp derivative strains. Bands corresponding to the 590 and 758 kb megaplasmids were visible in the undigested DNA patterns of the WT strains ISS-21 and ISS-190, respectively, whereas they were absent from the undigested DNA patterns of the respective derivative strains (hereafter designated as ISS-21Δ*mp590* and ISS-190Δ*mp758*, respectively), confirming successful megaplasmid removal in the latter strains. On the contrary, the smaller plasmid band was visible in the undigested DNA patterns of the WT strains as well as in those of the Δmp derivative strains, indicating that the smaller plasmid was present in the Δmp strains. However, the PFGE analysis did not allow us to identify other mutations possibly induced by acridine orange in the DNA of the Δmp strains.

**FIGURE 2:**
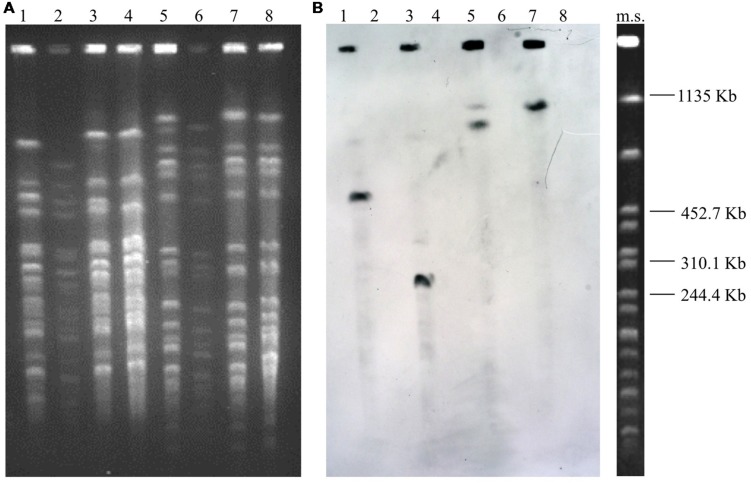
**Pulsed-field gel electrophoresis patterns of the genomic DNA of the WT *C. butyricum* type E strains and their Δmp derivative strains restricted with *XhoI* (lanes 1–4) and *SmaI* (lanes 5–8) **(A)**, and Southern hybridization analysis using the *latt* gene probe **(B)**.** Strains ISS-21 (lanes 1 and 5); ISS-21Δ*mp590* (lanes 2 and 6); ISS-190 (lanes 3 and 7); ISS-190Δ*mp758* (lanes 4 and 8). Ms, molecular standard, *Xba*I-digested DNA from *Salmonella* Braenderup strain H9812 (Hunter et al., 2005).

In the Southern blot experiments, the *bont/e* and *mob* gene probes hybridized to the chromosome and smaller plasmid bands, respectively, of all WT and Δmp strains (**Figures 1B,D**). The DNA probe targeting the megaplasmid-encoded beta-lactamase (*latt*) gene hybridized to the undigested and digested DNA of the WT strains ISS-21 and ISS-190; conversely, they failed to hybridize to either the undigested or digested DNA of the derivatives ISS-21Δ*mp590* and ISS-190Δ*mp758*, thus confirming that the latter strains lacked the megaplasmid-encoded beta-lactamase genes, which is consistent with the hypothesis that they had been cured of their megaplasmids (**Figures 1C** and **2**).

### Expression of BoNT/E

To assess the difference between the transcription rates of the chromosomal *bont/e* gene in the WT and Δmp derivative strains, the relative mRNA levels of *bont/e* were compared for each strain during the mid-exponential, late-exponential, early stationary, and mid-stationary growth phases.

**FIGURE 3:**
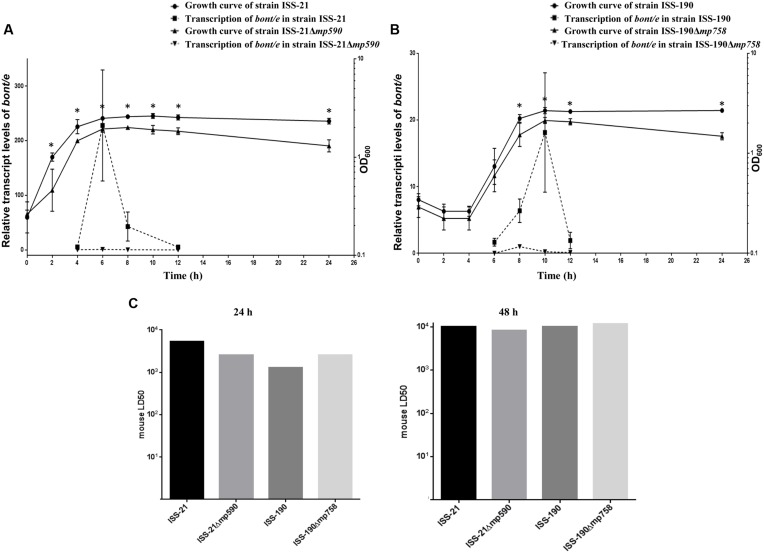
**Growth curves and relative transcript levels of *bont/e* of strains ISS-21 and ISS-21Δ*mp590***(A)** and of strains ISS-190 and ISS-190Δ*mp758***(B)**.** The *bont/e* transcript levels were normalized to the 16S *rrn* transcript level. Plotted values are means of three replicates. BoNT/E toxin levels expressed as mouse LD50 in the supernatants of 24 and 48 h TPGY cultures of the WT and Δmp derivative strains **(C)**. ^∗^*P* < 0.05.

Similar *bont/e* transcription curves as a function of time were observed in the WT strains ISS-21 and ISS-190; in fact, the relative transcript levels of *bont/e* were low during the mid-exponential phase, reached a maximum in the transition between the exponential and stationary phases, and then rapidly declined during the mid-stationary phase (**Figures 3A,B**), as was previously observed for *C. botulinum* strains of different types (Bradshaw et al., 2004; Chen et al., 2008; Connan et al., 2013).

Conversely, the relative transcript levels of *bont/e* in the Δmp derivative strains remained consistently low at all tested time points (**Figures 3A,B**). Specifically, the maximum transcript levels of *bont/e* were up to 240-fold lower in the cured strain ISS-21Δ*mp590* than in the parental strain ISS-21 (*p* < 0.01; **Figure 3A**), and up to 18-fold lower in the cured strain ISS-190Δ*mp758* than in the parental strain ISS-190 (*p* < 0.05; **Figure 3B**).

To investigate whether megaplasmid curing affected the production of BoNT/E, the toxicity of trypsin-treated supernatants from the cultures of the WT and Δmp strains was measured by a mouse bioassay after 24 and 48 h of culture, when the highest botulinum toxin levels should be present (Bradshaw et al., 2004; Couesnon et al., 2006). The results of the mouse bioassay revealed that the toxicity levels of the WT and Δmp strains supernatants were similar (approximately 10^3^ mouse LD50/ml after 24 h culture and 10^4^ mouse LD50/ml after 48 h culture; **Figure 3C**).

### Growth Studies Under Favorable and Limiting Environmental Conditions

The growth of the WT and Δmp derivative strains was first monitored at OD_600_ under favorable environmental conditions, with TPGY broth (pH 7) as the growth medium and incubation at 37°C under anaerobic atmosphere (Hatheway, 1992). In these conditions, both the WT strains (ISS-21 and ISS-190) reached similar maximum growth peak values after approximately 10 h of incubation (**Figures 3A,B**). A longer lag phase (4 h vs. <2h) and a faster exponential phase (6 h vs. ∼10 h) were observed in the growth curve of the WT strain ISS-190 compared to ISS-21. Under the same favorable environmental conditions, the growth rates of the Δmp derivative strains were significantly lower compared to their WT parental strains, reaching lower ODs at each tested time point, including the maximum growth peaks after 10 h of incubation (**Figures 3A,B**).

We then compared the growth of the WT and Δmp derivative strains under the specific limiting conditions of pH (5.0), NaCl concentration (3%), and incubation temperature (15°C), which are values that are close to the minimum pH, salinity, and temperature values reported for neurotoxigenic *C. butyricum* type E, respectively (Peck, 2002). As expected, under each limiting condition, both cured and uncured strains grew more slowly than under the optimal conditions; consequently, the experimental time periods were extended to 8 days under the limiting conditions of pH and salinity, and to 20 days when strains were incubated at 15°C.

**FIGURE 4:**
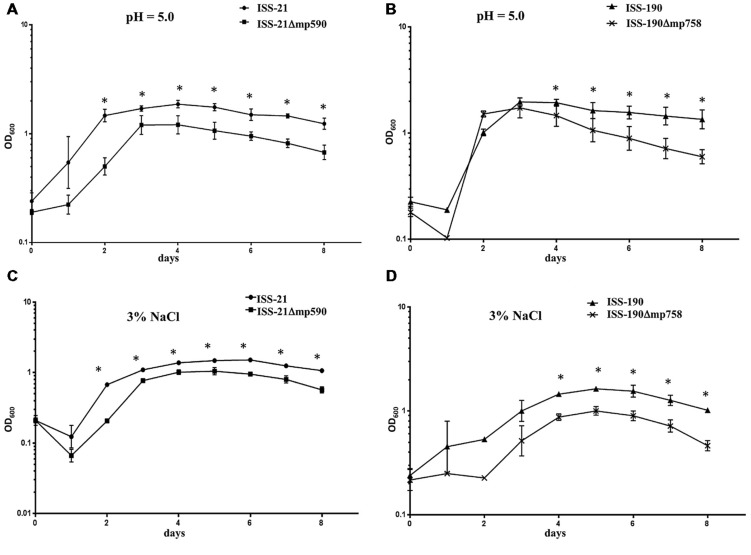
**Growth curves of WT and Δmp derivative strains at pH 5 **(A,B)** or in the presence of 3% NaCl **(C,D)**.** Plotted values are means of three replicates. Error bars represent the standard deviations of the mean. ^∗^*P* < 0.05.

Similar results were observed when the growth curves of the strains were compared at pH 5 (**Figures 4A,B**) or in the presence of 3% NaCl (**Figures 4C,D**). Under both experimental conditions, each Δmp derivative strain showed ODs lower than those of their respective WT parental strain, and the differences became statistically significant when they entered the exponential phase and at all subsequent time points throughout the 8 days duration of the experiments (**Figure 4**). The duration of the growth phases were similar for the WT and Δmp derivative strains: specifically, all cured and uncured strains reached their maximum growth peak between days 3 and 4 at pH 5, and between days 5 and 6 in the presence of 3% NaCl (**Figure 4**).

**FIGURE 5:**
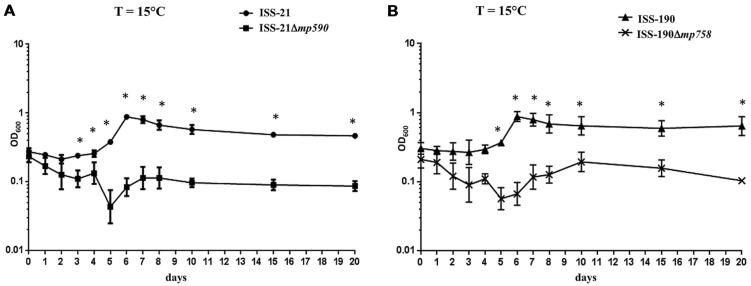
**Growth curves at 15°C of strains ISS-21 and ISS-21Δ*mp590* (A); and ISS-190 and ISS-190Δ*mp758* (B).** Plotted values are means of three replicates. Error bars represent the standard deviations of the mean. ^∗^*P* < 0.05.

**Figure 5** shows the growth curves of the WT and Δmp derivative strains, respectively, after incubation at 15°C for 20 days. Both the WT strains ISS-21 and ISS-190 exhibited a lag phase of approximately 4 days, after which they entered the exponential phase until reaching their maximum growth peak at day 6; the ODs of both WT strains then declined (mortality phase). Notably, no increase was observed in the ODs of either the ISS-21Δ*mp590* or ISS-190Δ*mp758* strains over the 20 days of the experiment, indicating that the Δmp derivative strains were not able to grow at 15°C.

### Beta-Lactamase Production, Sugar Fermentation Patterns and Spore Production

Neither the WT nor Δmp derivative strains displayed any beta-lactamase activity, with or without induction; in fact, exposure of the strains to beta-lactams did not induce beta-lactamase production. Growth of the strains only occurred in the presence of very low maximum concentrations of penicillin or ampicillin (0.125 and 0.062 μg/ml, respectively), with no detectable difference between the antibiotic susceptibilities of the WT and Δmp derivative strains.

The results of the sugar fermentation assays showed that the WT *C. butyricum* type E strains ISS-21 and ISS-190 fermented all the 14 carbohydrates analyzed; on the contrary, both the cured derivatives, ISS-21Δ*mp590* and ISS-190Δ*mp758*, were unable to ferment five carbohydrates, namely arabinose, glycerol, mannitol, rhamnose, and xylose.

After 20 days of anaerobic incubation at 37°C all analyzed strains produced spores. Specifically, both WT strains (ISS-21 and ISS-190) produced ∼10^2^ spores/ml each, whereas their derivatives (ISS-21Δ*mp590* and ISS-190Δ*mp758*) produced ∼10^4^ spores/ml each; i.e., the Δmp derivative strains yielded nearly 2 logs more viable spores than their WT parental strains.

## Discussion

In the present study, we demonstrated that two neurotoxigenic *C. butyricum* type E strains could be cured of their megaplasmids of 590 and 758 kb, respectively, without loss of viability, in accordance with the general definition of plasmids as dispensable genetic elements. Megaplasmid curing was achieved by chemical treatment with acridine orange, which is also known to cause frameshift mutations; however, these are very small changes compared with loss of megaplasmids which constitute 13–19% of the whole bacterial genome (Franciosa et al., 2011), hence we assumed that the observed effects in the cured strains were primarily caused by the megaplasmid loss. Spontaneous loss of megaplasmids did not occur and another curing strategy, i.e., repeated sub-culturing at 44°C, a method previously used for curing the plasmids of *C. botulinum* strains (Eklund et al., 1988), did not work (data not shown). These observations imply that the megaplasmids are inherently stable in their microbial hosts, further supporting the existence of essential reasons other than viability for the maintenance of the megaplasmids.

Since the most significant characteristic of the neurotoxigenic *C. butyricum* type E strains is their ability to produce BoNT/E, we first investigated whether the megaplasmids affected expression of BoNT/E, a property that had not yet been documented in the peculiar neurotoxigenic *C. butyricum* type E strains. Our RTq-PCR results showed significantly higher amounts of *bont/e* mRNA in both the WT strains than in their Δmp derivative strains, and that the difference between the transcript levels of *bont/e* was more pronounced when the strain possessing the smaller megaplasmid was compared to its cured derivative. These results indicate that transcriptional activators of the chromosomal *bont/e* gene are likely present in the genetic region common to the differently sized megaplasmids, while the 168 kb genetic region unique to the larger megaplasmid possibly harbors down-regulators of *bont/e* gene transcription. *In silico* analysis of the genetic region common to the megaplasmids revealed that it contains several putative transcriptional regulatory elements, including a sigma factor and six two-component systems (TCSs), whereas five additional TCSs and a CRISPR-cas locus that has also been demonstrated to play regulatory roles in some bacteria (Sampson and Weiss, 2014) are located in the 168 kb genetic region only present in the larger megaplasmid.

Most notably, however, we found that the variation in the transcript levels of *bont/e* did not reflect variation in the levels of BoNT/E produced by strains after 24 and 48 h culture: in fact, despite the significantly higher transcript levels of the *bont/e* gene in the WT strains compared to the Δmp derivative strains, BoNT/E production as tested by the mouse bioassay did not substantially differ between the WT and Δmp derivative strains. These findings suggest that the megaplasmids do not ultimately control the BoNT/E production; besides, they could indicate that currently unknown downstream controls at the post-transcriptional or translational levels and/or on protein folding, secretion and degradation could exert a major influence on the expression of BoNT/E by the neurotoxigenic *C. butyricum* strains. The latter deduction would be in line with the accumulating evidence that levels of control occurring after mRNA formation play a more significant role in the regulation of protein formation than previously appreciated (Foss et al., 2011; Vogel and Marcotte, 2012). However, this hypothesis will need to be confirmed by comparing the kinetic patterns of BoNT/E production by the WT and cured strains.

Relatively little is known about the regulation of BoNT production in neurotoxigenic clostridia strains. Expression of BoNT/A in the *C. botulinum* strain Hall, which is the most studied strain in this field, has been shown to be mainly regulated at the transcriptional level by an alternative sigma factor encoded by the *botR* gene and by several TCSs (Connan et al., 2013; Connan and Popoff, 2015). Nevertheless, the BoNT/E-producing *C. butyricum* and *C. botulinum* strains lack *botR* gene homologs in their genomes, suggesting the existence of alternative regulatory strategies for BoNT/E expression in these strains (Connan et al., 2013).

Another major finding of this study was that growth of the neurotoxigenic *C. butyricum* type E strains cured of their megaplasmids was totally impaired at the moderately low temperature of 15°C, clearly indicating that both the differently sized megaplasmids are essential for the adaptation of their hosts to low temperatures. Different TCSs have been involved in cold tolerance and adaptation of *C. botulinum* strains (Lindström et al., 2012; Derman et al., 2013; Mascher et al., 2014); thus, it may be interesting to assess whether one or more of the TCSs of the megaplasmids, particularly those encoded by the shared genetic region, play(s) similar roles in the neurotoxigenic *C. butyricum* type E strains. Although the environmental source of the clinical *C. butyricum* type E strains used in this study was not originally identified, other neurotoxigenic *C. butyricum* type E strains have been isolated from lake sediments and aquatic animals (Meng et al., 1999; Shelley et al., 2015), suggesting that these microorganisms are widespread in aquatic environments, as for the *C. botulinum* type E strains (Horowitz, 2010; Espelund and Klaveness, 2014). Our findings are consistent with those of a recent study that predicted an adaptive role for plasmids of psychrophilic and psychrotolerant bacteria based on *in silico* meta-analysis of the plasmid sequences (Dziewit and Bartosik, 2014). Of note, the ability of the *C. butyricum* type E strains to grow at 15°C has also impacts on food safety issues, for instance if temperature abuse occurs during storage.

In addition, we found that growth of the Δmp neurotoxigenic *C. butyricum* type E strains was significantly lower than for the WT strains at optimal environmental conditions, as well as under limiting conditions of pH and salt concentrations. The loss of such large plasmids likely results in pleiotropic effects, thus impairing metabolism and modifying the physiologic efficiency of the host bacteria.

Our finding that the strains lacking the megaplasmids were unable to ferment several carbohydrates, including glycerol, confirms that the megaplasmids play a role in multiple metabolic pathways; this is also supported by the fact that the annotated sequence of the megaplasmid (contig 1 of GenBank Reference NZ_ACOM00000000.1) contains a number of genes for sugar metabolism, including a complete 1,3-propanediol operon (9182 bp in length) potentially involved in the fermentation of glycerol.

Interestingly, under identical conditions, larger spore numbers were produced by the Δmp derivative strains than by their WT parental strains. Since the Δmp strains are less fit under certain environmental conditions, as discussed above, they may have developed greater spore-forming ability in order to survive; however, this remains a speculative hypothesis, and further research is needed on the regulatory mechanisms controlling sporulation in these strains.

Finally, we compared the WT and Δmp derivative strains for beta-lactamase production. We had previously hypothesized that the megaplasmids may confer resistance to beta-lactam antibiotics mediated by beta-lactamases to their microbial hosts because the genetic region common to the larger and smaller megaplasmids contains at least four putative beta-lactamase genes (Franciosa et al., 2011; Iacobino et al., 2013). However, our present results showed that neither the WT nor Δmp derivative strains produced beta-lactamases, even after induction, and that all cured and uncured strains were highly susceptible to penicillin G and ampicillin, i.e., two of the most widely used beta-lactam antibiotics. Thus, these results indicate that either the megaplasmid-encoded beta-lactamase genes are inactive because of mutations or their expression is repressed.

## Conclusion

We have demonstrated that the megaplasmids of neurotoxigenic *C. butyricum* type E strains are not related to beta-lactam resistance, and nor do they ultimately control the synthesis of BoNT/E; rather, they encode functions that are relevant for the growth of their hosts under optimal and limiting environmental conditions, and that are critical for growth at low temperatures. Future efforts should focus on the characterization of the megaplasmid genes and their impact on specific phenotypes as well as on the megaplasmids mobility properties, transfer efficiencies and host range, in order to decipher their role in the emergence and spread of their microbial hosts.

## Author Contributions

GF and SM designed the study. CS, AI, and LG performed the experiments. GF, SM, AI, and CS contributed to data analysis. GF drafted the manuscript. All authors reviewed and approved the final manuscript.

## Conflict of Interest Statement

The authors declare that the research was conducted in the absence of any commercial or financial relationships that could be construed as a potential conflict of interest.
